# Geographic Variation in Mortality of Acute Myocardial Infarction and Association With Health Care Accessibility in Beijing, 2007 to 2018

**DOI:** 10.1161/JAHA.123.029769

**Published:** 2023-06-10

**Authors:** Jie Chang, Qiuju Deng, Piaopiao Hu, Moning Guo, Feng Lu, Yuwei Su, Jiayi Sun, Yue Qi, Ying Long, Jing Liu

**Affiliations:** ^1^ Center for Clinical and Epidemiologic Research Beijing An Zhen Hospital, Capital Medical University, Beijing Institute of Heart, Lung and Blood Vessel Diseases, National Clinical Research Center of Cardiovascular Diseases, Beijing Municipal Key Laboratory of Clinical Epidemiology Beijing China; ^2^ Beijing Municipal Health Big Data and Policy Research Center Beijing Institute of Hospital Management Beijing China; ^3^ School of Urban Design Wuhan University Wuhan China; ^4^ School of Architecture and Hang Lung Center for Real Estate, Key Laboratory of Eco Planning & Green Building, Ministry of Education Tsinghua University Beijing China

**Keywords:** acute myocardial infarction, geographic variation, health care accessibility, mortality, Epidemiology, Myocardial Infarction, Health Services

## Abstract

**Background:**

Little is known about geographic variation in acute myocardial infarction (AMI) mortality within fast‐developing megacities and whether changes in health care accessibility correspond to changes in AMI mortality at the small‐area level.

**Methods and Results:**

We included data of 94 106 AMI deaths during 2007 to 2018 from the Beijing Cardiovascular Disease Surveillance System in this ecological study. We estimated AMI mortality for 307 townships during consecutive 3‐year periods with a Bayesian spatial model. Township‐level health care accessibility was measured using an enhanced 2‐step floating catchment area method. Linear regression models were used to examine the association between health care accessibility and AMI mortality. During 2007 to 2018, median AMI mortality in townships declined from 86.3 (95% CI, 34.2–173.8) to 49.4 (95% CI, 30.5–73.7) per 100 000 population. The decrease in AMI mortality was larger in townships where health care accessibility increased more rapidly. Geographic inequality, defined as the ratio of the 90th to 10th percentile of mortality in townships, increased from 3.4 to 3.8. In total, 86.3% (265/307) of townships had an increase in health care accessibility. Each 10% increase in health care accessibility was associated with a −0.71% (95% CI, −1.08% to −0.33%) change in AMI mortality.

**Conclusions:**

Geographic disparities in AMI mortality among Beijing townships are large and increasing. A relative increase in township‐level health care accessibility is associated with a relative decrease in AMI mortality. Targeted improvement of health care accessibility in areas with high AMI mortality may help reduce AMI burden and improve its geographic inequality in megacities.

**Nonstandard Abbreviations and Acronyms**
BCDSS: Beijing Cardiovascular Disease Surveillance System

**Clinical Perspective**

**What Is New?**

- Using citywide small‐area data from Beijing, we found that acute myocardial infarction mortality and its change over time varied considerably across townships.
- A relative increase in township‐level health care accessibility is associated with a relative decrease in acute myocardial infarction mortality.

**What Are the Clinical Implications?**

- Our findings advance the understanding of regional health disparities within rapidly developing megacities.
- Localized policy actions aimed at improving health care accessibility in areas with high acute myocardial infarction mortality may help reduce acute myocardial infarction mortality and improve its geographic inequality in megacities.

Ischemic heart disease is a leading cause of death worldwide.^1^ Acute myocardial infarction (AMI) is a serious manifestation of ischemic heart disease and is potentially fatal in the absence of appropriate intervention.^2^ Information on how mortality varies geographically at the small‐area level could inform targeted and equitable health policies toward the goal of improving population health for all.^3^ Prior studies in developed countries have found small‐area variation in AMI mortality within cities and have highlighted priorities for intervention efforts^4^, ^5^, ^6^, ^7^; however, limited data are available for developing countries.

Beyond identifying geographic variation in AMI mortality, exploring factors associated with areas that have high mortality may provide important insights into strategies to control the disease burden. Access to health care is one of the most important social determinants of cardiovascular health.^8^ Previous studies have used driving time or distance to a hospital as indicators of health care accessibility and reported the adverse effects of a longer driving time/distance to the hospital on AMI mortality.^9^, ^10^ However, the driving time/distance does not take into account health care demand volume and health care supply capacity. A cross‐sectional study using the 2‐step floating catchment area method, which incorporates the interaction between health care demand and supply, explored the association between access to cardiac diagnostic testing and acute coronary syndrome mortality.^11^ However, whether changes in health care accessibility correspond to changes in AMI mortality at the small‐area level, which could provide benchmarks for future health policy decisions, remains unclear.

Health care accessibility is potentially crucial in populous cities. Beijing, China's capital, is one of the most populous megacities in the world. In the past decade, this city has experienced rapid population growth and improved health care.^12^ However, the high quality of health care resources greatly varies among different areas, and most high‐quality health resources cluster in urban areas of the city.^13^ With its highly heterogeneous health care environments, Beijing provides an ideal setting to investigate the geographic variations in AMI mortality at a finer spatial resolution and to evaluate their association with health care accessibility.

In this study, using data on AMI deaths extracted from the Beijing Cardiovascular Disease Surveillance System (BCDSS) from 2007 to 2018, we quantified geographic variations in AMI mortality at the township level in Beijing. Furthermore, we examined whether changes in health care accessibility are associated with changes in AMI mortality at this spatial scale.

## METHODS

The data used in this study were obtained from the Beijing Municipal Health Big Data and Policy Research Center and cannot be shared publicly, given institutional regulations and data confidentiality agreements. These data may be requested by researchers from the above data holder authorities for research purposes. The analytic methods can be reproduced based on the details provided in this article, and the statistical analysis code is available upon request. The corresponding author (J.L.) has full access to all of the data used in the study and takes full responsibility for its integrity and the data analysis. The study was approved by the ethics review committee of Beijing An Zhen Hospital, Capital Medical University, with a waiver of informed consent (2021139X).

### Study Setting

Beijing covers a total area of 16 410 km^2^ and comprises 6 districts located in the city's urban core and 10 districts in periurban areas (Figure S1). Districts can be further divided into townships, the smallest administrative unit in China. We conducted a geographic analysis at the township level. The number of townships in Beijing varied from 317 to 333 between 2007 and 2018. Given the changing administrative boundaries of some townships over time and to ensure consistency between each township population and the township boundaries, we combined some townships to ensure stable units of analysis over the study period, giving a stable number of townships (n=307) for the analysis (Table S1). The median permanent population of a township was 33 903, with 10th and 90th percentile populations of 10 614 and 86 895, respectively.

### Data Sources

We identified AMI deaths in Beijing using the BCDSS, which links routinely collected records in the Beijing Hospital Discharge Information System and the Beijing Vital Registration Monitoring System using personal identification information. The Hospital Discharge Information System of the Beijing Municipal Health Big Data and Policy Research Center covers admissions to all secondary‐ and tertiary‐level hospitals in Beijing.^14^ The Vital Registration Monitoring System of the Beijing Center for Disease Prevention and Control covers all deaths in Beijing.^15^ Deaths from AMI were identified according to the underlying cause of death using *International Classification of Diseases, Tenth Revision* (*ICD‐10*) codes I21 to I22 (acute myocardial infarction and subsequent myocardial infarction).^4^ Additionally, 2738 AMI deaths that were identified in the Hospital Discharge Information System but not in the Vital Registration Monitoring System were also included in our study. Finally, a total of 94 106 AMI deaths between 2007 and 2018 among permanent Beijing residents aged ≥35 years were included in this analysis. The diagnosis of AMI in the BCDSS has been validated, as described in Data S1. Information on the residential address for each AMI death was obtained from the BCDSS. To protect patient privacy, the number of the smallest unit for the address, such as the apartment number, was deleted in the database. Patient addresses were geocoded to latitude and longitude coordinates and then geographically aggregated within townships.

Annual population data from 2007 to 2018 by sex at the township level were extracted from the statistical yearbooks for districts issued by the Beijing Municipal Bureau Statistics. This resulted in the study analyses being performed using township as the sampling unit and not individuals. Because population data stratified by age and sex are only reported at the district level, the township population by age and sex was estimated by applying the district level population distributions. The population estimation method has been described in our previous study, which also used the population distributions of the district‐level spatial unit to estimate population distributions of the township‐level spatial unit.^16^

### Health Care Accessibility Index

Health care accessibility at the township level was measured using an enhanced 2‐step floating catchment area method based on a Gaussian function. This method incorporates the interaction between health care supply and potential demand.^17^ The Gaussian function is adopted to model the distance‐decay effects as suggested in an existing study,^17^ which assumes that even within the same catchment area (maximum service area of a hospital), people would give preference to closer hospitals over hospitals that are farther away, and with longer driving time to a hospital, the less likely the hospital will be selected. The enhanced 2‐step floating catchment area method measures accessibility in 2 steps. The first step is calculation of the supply‐to‐demand ratio within a driving time threshold (maximum service area of each hospital). The second step involves summing the supply‐to‐demand ratios of all hospitals within the driving time threshold of each township. In line with previous research, we set the number of hospital beds as the health care supply capacity.^17^ The demand size refers to the number of township populations covered by hospital service areas. The health care accessibility value is equal to the number of hospital beds per 1000 population. We also used the number of health care personnel as the health care supply capacity in sensitivity analysis. Details of the enhanced 2‐step floating catchment area method are described in Data S2. We further divided hospitals into percutaneous coronary intervention (PCI) hospitals and non‐PCI hospitals and measured PCI‐hospital accessibility and non–PCI‐hospital accessibility. Hospitals in the BCDSS that reported performing PCI for patients with AMI were considered PCI hospitals.

### Covariates

Socioeconomic factors included average per capita disposable income from 2015 to 2018 at the district level obtained from the statistical yearbook,^18^ the proportion of the population with a high school education or above at the township level, and the proportions of unemployed and of married people in the population at the district level extracted from the 2010 population census.^19^ Cardiovascular risk factors at the district level included the prevalence of hypertension, diabetes, hypercholesterolemia, and smoking in 2008, 2011, 2014, and 2017 (Table S2).

### Statistical Analysis

#### Calculation of AMI Mortality at Township Level

AMI mortality estimates in small areas such as townships may be unstable owing to small numbers of deaths. We aggregated AMI deaths and populations into four 3‐year periods (2007–2009, 2010–2012, 2013–2015, and 2016–2018) to ensure sufficient numbers of events for stable estimates at fine scale. Then, township AMI mortality was estimated separately for men and women, each age group (35–64 years, 65 to 79 years, and ≥80 years), and each 3‐year period, using the Bayesian spatial model. The model, known as the Besag, York, and Mollie model,^20^ where information is shared locally (ie, among adjacent townships) through spatially structured random effects with a conditional autoregressive prior and globally through spatially unstructured Gaussian random effects. This approach balances overly unstable within‐township estimates and overly simplified aggregate large‐area estimates that mask small‐area variation. Details of the model are described in Data S3.

Age‐standardized mortality rates for men and women were calculated by the direct standardization method using the Beijing population obtained from the 2010 national population census as the standard population. The corresponding weights were 82.9%, 14.0%, and 3.1% for the age groups of 35 to 64, 65 to 79, and ≥80 years for standardization, respectively. In addition, age‐ and sex‐standardized mortality rates for the total population were also calculated through the direct standardization method using the 2010 Beijing population as the standard population. Age and sex categories for standardization included men and women aged 35 to 64, 65 to 79, and ≥80 years, with corresponding weights of 43.1%, 6.6%, and 1.4% for the 3 age groups in men, and 39.8%, 7.4%, and 1.7% for those in women, respectively. We reported the posterior mean and 95% CI of AMI mortality for each township. We measured the geographic inequalities in AMI mortality among townships using the ratio of the 90th to 10th percentiles of AMI mortality in townships, according to the literature.^21^ To match the period of AMI mortality, we calculated the township health care accessibility for the corresponding 3‐year period from 2007 to 2018.

#### Analyses of Association Between Health Care Accessibility and AMI Mortality

A mixed linear model accounting for longitudinal data was used to explore the association between township health care accessibility and AMI mortality, including a random intercept of townships. The estimated coefficients (β) and corresponding 95% CIs were reported. Three models were constructed. Model 1 was univariate. Model 2 was adjusted for the prevalence of hypertension, diabetes, hypercholesterolemia, and smoking. Model 3 was further adjusted for the proportion of married in the population, the proportion of the population with a high school education or above, per capita disposable income, and proportion of unemployed in the population.

Linear regression models were fitted with percentage change in AMI mortality from 2007 to 2018 as the dependent variable and percentage change in health care accessibility from 2007 to 2018 as independent variables to assess the association between the changes in health care accessibility and changes in AMI mortality.

Two sensitivity analyses were performed to check robustness of the results. First, we measured geographic inequality in AMI mortality among townships using the coefficient of variation. We calculated the coefficient of variation because it uses the full distribution of township mortality rates to measure the amount of geographic inequality rather than relying on the tails of the distribution to calculate the disparity ratio.^22^ A large coefficient of variation indicates more inequalities in AMI mortality among townships. Second, we used the number of health care personnel instead of hospital beds as the health care supply capacity to calculate health care accessibility and further evaluated the association of health care accessibility with AMI mortality.

Data analyses were performed using R software, version 4.0.0 (R Foundation for Statistical Computing, Vienna, Austria), and ArcGIS software, version 10.5 (ESRI, Redlands, CA). A 2‐sided value *P*<0.05 was considered statistically significant.

## RESULTS

### Temporal Trends in AMI Mortality

A total of 94 106 AMI deaths occurred among permanent residents of Beijing aged ≥35 years between 2007 and 2018, with 45.1% women. At the township level, the mean age of patients with AMI during 2007 to 2018 was 74.8 years (SD, 2.4; range 67.2–80.2 years). After age and sex standardization, the median AMI mortality declined from 86.3 (95% CI, 34.2–173.8) to 49.4 (95% CI, 30.5–73.7) per 100 000 population from 2007 to 2018, a relative decline of 42.8%. The median mortality rate decreased 38.8% for men, from 98.9 (95% CI, 60.2–150.6) to 60.5 (95% CI, 32.4–100.8) per 100 000 population, and the rate for women decreased 47.3%, from 70.6 (95% CI, 46.1–100.8) to 37.2 (95% CI, 24.8–53.2) per 100 000 population (Table 1).

**Table 1:** Number of Deaths and Age‐Standardized Mortality of Acute Myocardial Infarction by Township in Beijing, 2007 to 2018

Characteristic	2007–2009		2010–2012		2013–2015		2016–2018	
	Deaths	Mortality/100 000	Deaths	Mortality/100 000	Deaths	Mortality/100 000	Deaths	Mortality/100 000
Total, median (IQR)	62 (36–97)	86.3 (61.3–127.8)	68 (38–107)	84.3 (59.4–130.0)	74 (36–106)	68.7 (50.2–114.9)	62 (31–93)	49.4 (39.4–80.7)
Men, median (IQR)	34 (19–53)	98.9 (72.4–150.5)	38 (20–60)	96.6 (72.2–149.2)	39 (19–58)	80.4 (60.3–131.4)	33 (17–52)	60.5 (48.9–97.3)
Women, median (IQR)	28 (14–43)	70.6 (49.5–106.9)	30 (16–48)	69.6 (45.1–104.2)	32 (16–48)	55.2 (37.7–92.9)	27 (13–42)	37.2 (28.7–60.2)

IQR indicates interquartile range.

### Geographic Patterns and Inequality in AMI Mortality

**Figure 1:**
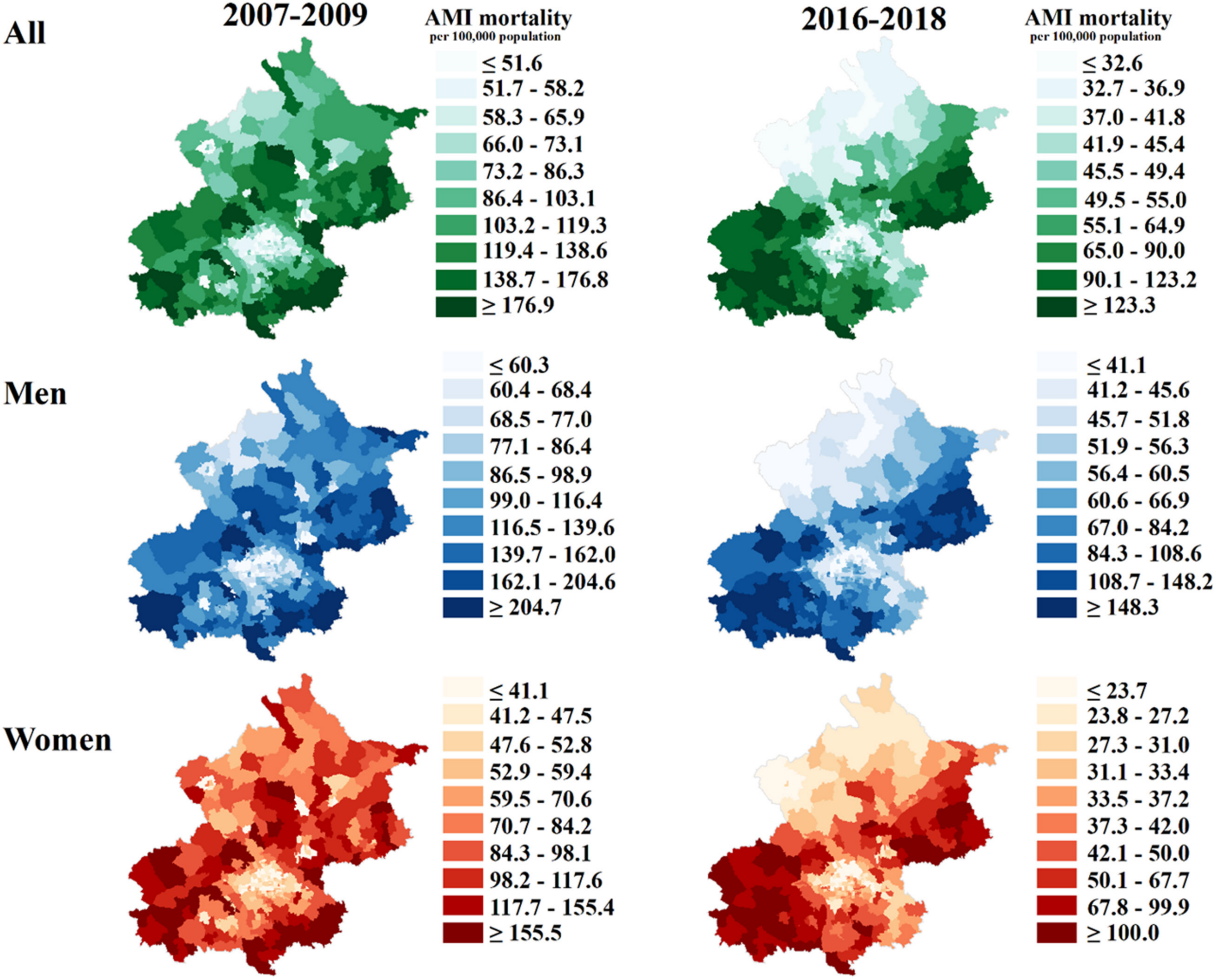
Deciles of age‐standardized mortality of acute myocardial infarction (AMI) in Beijing residents aged ≥35 years at township level, 2007 to 2018. The left map shows the mortality of AMI at the beginning of the study period (2007–2009), and the right map shows the mortality at the end of the study period (2016–2018). The green, blue, and red maps indicate the mortality in the total population, men, and women, respectively. A darker color indicates a higher mortality.

In 2007 to 2009, townships with high AMI mortality (in the top decile) were scattered throughout periurban areas of Beijing, whereas townships with low AMI mortality (in the bottom decile) were concentrated in urban core areas. In 2016 to 2018, townships with high AMI mortality were highly clustered in southwestern and northeastern periurban areas, whereas townships with low AMI mortality remained predominantly in urban core areas, with some emerging in northern periurban areas. For men and women, the geographic patterns in AMI mortality were similar to those of the general population during the study period (Figure 1). The geographic patterns of AMI mortality from 2010 to 2015 are shown in Figure S2. For men, the highest proportion of deaths was observed in those aged 65 to 79 years (median [interquartile range {IQR}], 38.4% [35.3%–42.4%]), followed by those aged ≥80 years (median [IQR], 33.5% [26.8%–40.8%]) and those aged 35 to 64 years (median [IQR], 26.9% [21.5%–33.0%]). For women, the highest proportion of deaths was observed in those aged ≥80 years (median [IQR], 50.4% [44.1%–57.7%]), followed by those aged 65 to 79 years (median [IQR], 38.7% [34.2%–43.8%]) and those aged 35 to 64 years (median [IQR], 10.0% [5.8%–14.6%]; Figure S3). In addition, the geographic patterns in AMI mortality by different age and sex groups during 2007 to 2018 were also presented in Figure S4 through S7. AMI mortality increased with age. For all age and sex groups, the geographic patterns in AMI mortality were similar to those of the general population for the corresponding study period.

Geographic inequality, defined as the ratio of the 90th to 10th percentiles of AMI mortality in townships, increased from 3.4 in 2007 to 2009 to 3.8 in 2016 to 2018. An increase in geographic inequality was also observed in men (3.4 to 3.6) and women (3.8 to 4.2).

### Geographic Patterns Within Changes in AMI Mortality

**Figure 2:**
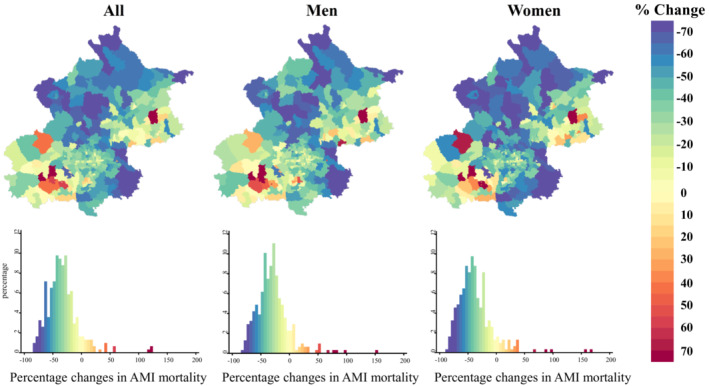
Percentage changes in age‐standardized mortality of acute myocardial infarction (AMI) in Beijing residents aged ≥35 years at township level, 2007 to 2018. The percentage change shows the relative change in the mortality of AMI from the beginning of the study period (2007–2009) to the end of the period (2016–2018). Blue indicates a decrease in mortality, and red indicates an increase in mortality. The histogram shows the distribution of the percentage change in the mortality of AMI for all townships.

From 2007 to 2018, 90.9% (279/307) of townships in Beijing experienced a decline in mortality. Furthermore, 25.7% (79/307) of townships declined by ≥50%, and more townships experienced a decline of ≥50% (35.8%) for women than men (20.9%). Townships with a rapid decline in mortality were located in the southeastern and northern periurban areas of Beijing, and the geographic patterns within these changes were similar for men and women (Figure 2).

### Association Between Health Care Accessibility and AMI Mortality

**Figure 3:**
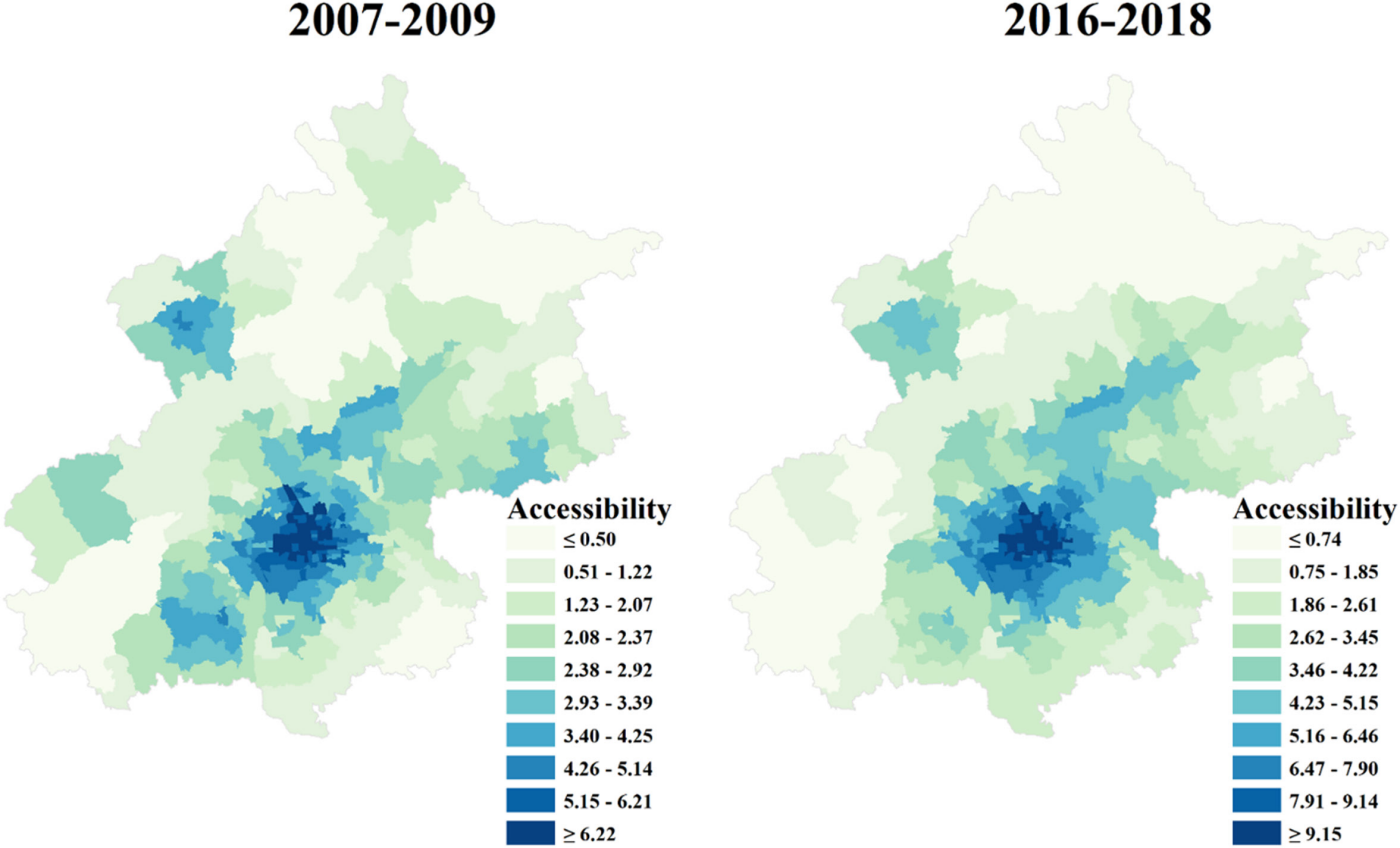
Deciles of health care accessibility in Beijing at township level, 2007 to 2018. The left and right maps show health care accessibility at the beginning of the study period (2007–2009) and the end of the study period (2016–2018), respectively. A darker color indicates a higher health care accessibility.

**Figure 4:**
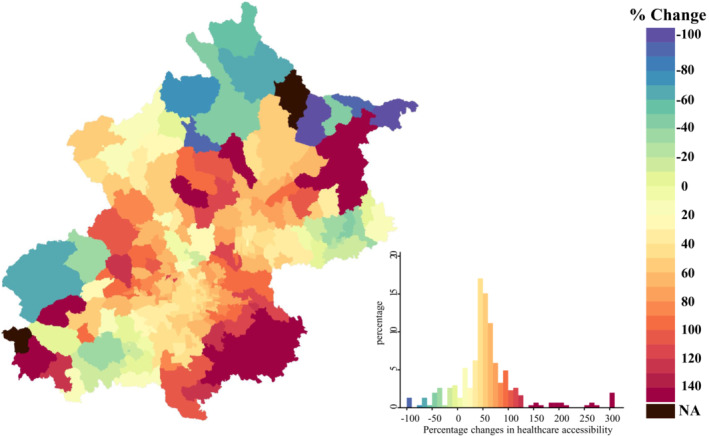
Percentage changes in health care accessibility in Beijing at township level, 2007 to 2018. The percentage change shows the relative change in health care accessibility from the beginning of the study period (2007–2009) to the end of the study period (2016–2018). Blue indicates a decrease in health care accessibility, and red indicates an increase in health care accessibility. The histogram shows the distribution of percentage changes in health care accessibility for all townships. NA indicates that the percentage changes in health care accessibility cannot be estimated, as the health care accessibility value of these townships is 0 at the beginning of the study period (2007–2009).

Townships with high health care accessibility were predominantly in urban core areas during the study period (Figure 3 and Figure S8). An increase in health care accessibility was widely observed in Beijing, with 86.3% (265/307) of townships experiencing an increase in health care accessibility from 2007 to 2018. Large increases were clustered in southeastern periurban areas and were also observed in northern periurban areas (Figure 4). Similar geographic patterns were observed for PCI‐hospital accessibility and non–PCI‐hospital accessibility (Figures S9 and S10).

Using univariate analysis, township‐level health care accessibility was negatively associated with AMI mortality (β, −12.47 [95% CI, −14.00 to −10.95]). The regression coefficient value was also robust to adjustments for cardiovascular risk factors (β, −10.96 [95% CI, −12.68 to −9.23]). After further adjustment for socioeconomic factors, associations were attenuated but remained directionally consistent (β, −6.47 [95% CI, −9.03 to −3.90]). Furthermore, this association was observed in both PCI‐hospital accessibility‐specific and non–PCI‐hospital accessibility‐specific analyses and was robust to all adjustments. In the fully adjusted analyses, both PCI‐hospital accessibility (β, −9.95 [95% CI, −13.76 to −6.41]) and non–PCI‐hospital accessibility were negatively associated with AMI mortality (β, −8.37 [95% CI, −13.70 to −3.03]; Table 2).

**Table 2:** Association Between Health Care Accessibility and Mortality of Acute Myocardial Infarction Among Beijing Townships

Accessibility	Model 1		Model 2		Model 3	
	β (95% CI)	*P* value	β (95% CI)	*P* value	β (95% CI)	*P* value
Total accessibility	−12.47 (−14.00 to −10.95)	<0.001	−10.96 (−12.68 to −9.23)	<0.001	−6.47 (−9.03 to −3.90)	<0.001
PCI‐hospital accessibility	−18.51 (−20.81 to −16.21)	<0.001	−15.94 (−18.41 to −13.47)	<0.001	−9.95 (−13.76 to −6.41)	<0.001
Non–PCI‐hospital accessibility	−25.43 (−29.30 to −21.57)	<0.001	−20.70 (−25.33 to −16.08)	<0.001	−8.37 (−13.70 to −3.03)	<0.001

Model 1: not adjusted. Model 2: adjusted for prevalence of hypertension+prevalence of diabetes+prevalence of hypercholesterolemia+prevalence of smoking. Model 3: model 2+proportion of married+proportion of population with high school education or above+per capita disposable income+proportion of unemployed. PCI indicates percutaneous coronary intervention.

We also explored the association between longitudinal changes in health care accessibility and changes in AMI mortality. After adjusting for cardiovascular risk factors, every 10% increase in health care accessibility was significantly associated with a −0.88% (95% CI, −1.28% to −0.49%) change in AMI mortality. After further accounting for socioeconomic factors, the effect estimates remained directionally consistent and stable (β, −0.71% [95% CI, −1.08% to −0.33%]). Furthermore, a relative increase in PCI‐hospital accessibility and non–PCI‐hospital accessibility was significantly associated with a relative decrease in AMI mortality in the fully adjusted analyses (β, −0.53% [95% CI, −0.96% to −0.11%]; and β, −0.35% [95% CI, −0.51% to −0.19%], respectively; Table 3).

**Table 3:** Association Between Percentage Changes in Health Care Accessibility and Percentage Change in Mortality of Acute Myocardial Infarction Among Beijing Townships

Accessibility	Model 1		Model 2		Model 3	
	β (95% CI)	*P* value	β (95% CI)	*P* value	β (95% CI)	*P* value
Per 10% increase						
Total accessibility	−0.01% (−0.22% to 0.21%)	0.954	−0.88% (−1.28% to −0.49%)	<0.001	−0.71% (−1.08% to −0.33%)	<0.001
PCI‐hospital accessibility	−0.69% (−1.11% to −0.28%)	0.001	−0.58% (−1.02% to −0.15%)	0.009	−0.53% (−0.96% to −0.11%)	0.014
Non–PCI hospital accessibility	−0.29% (−0.46% to −0.12%)	0.001	−0.46% (−0.62% to −0.30%)	<0.001	−0.35% (−0.51% to −0.19%)	<0.001

Model 1: not adjusted. Model 2: adjusted for percentage change in prevalence of hypertension+percentage change in prevalence of diabetes+percentage change in prevalence of hypercholesterolemia+percentage change in prevalence of smoking. Model 3: model 2+proportion of married+proportion of population with high school education or above+per capita disposable income+proportion of unemployed. PCI indicates percutaneous coronary intervention.

### Sensitivity Analyses

The increase in geographic inequality in AMI mortality among townships during the study period was also observed in the sensitivity analyses when using the coefficient of variation to measure geographic inequality (Table S3). The association of health care accessibility with AMI mortality was similar to the main analyses when using the number of health care personnel instead of hospital beds as the health care supply capacity to calculate health care accessibility (Tables S4 and S5).

## DISCUSSION

### Main Findings

Using citywide data from Beijing, we found that nine‐tenths of townships experienced a decrease in AMI mortality over the past decade. However, the size of the decline in AMI mortality varied considerably across townships; large and increasing geographic inequalities (a >3‐fold difference) in AMI mortality were observed. Improvements in health care accessibility were widely observed in Beijing townships. Furthermore, township‐level health care accessibility was negatively associated with AMI mortality, and a relative increase in health care accessibility was associated with a relative decrease in AMI mortality.

### Comparisons With Other Studies

Prior studies from developed countries have identified geographic inequalities in AMI mortality at the small‐area level within cities.^4^, ^5^, ^6^, ^7^ Consistent with previous research, we also found substantial geographic disparities in AMI mortality at high spatial resolution in Beijing. To the best of our knowledge, small‐area geographic variations in AMI mortality have not been studied previously within populous, fast‐developing megacities. Research conducted at the province level (with Beijing as a province) in China has shown that the geographic inequality in ischemic heart disease mortality was 2.8 in 2016.^23^ The level of inequality in AMI mortality (a 3.8‐fold difference in 2016–2018) among Beijing townships was greater than that of ischemic heart disease mortality among Chinese provinces.^23^ Our study revealed within‐city variation in China that may be masked by city‐level averages.

Our findings showed that township‐level health care accessibility was negatively associated with AMI mortality. These findings are concordant with previous studies that highlight the adverse effects of poor health care accessibility on AMI mortality at the block group level in the United States^10^ and the secondary medical service area level^24^ and prefecture level^25^ in Japan. Furthermore, our study adds to these findings in that a relative increase in health care accessibility was longitudinally associated with a relative decrease in AMI mortality.

### Interpretation of Results

The geographic variations in AMI mortality observed in our study may be attributed to variations in the prevalence of risk factors and levels of health care. In Beijing, the prevalence of risk factors tends to be higher where AMI mortality is high. For example, the Beijing Adult Tobacco Survey showed that the prevalence rate of smoking in 2019 was 19.2% among urban residents and 24.7% among periurban residents,^26^ which may partly be associated with the lower AMI mortality in urban townships than in periurban townships. Moreover, recent studies found a higher prevalence of risk factors (eg, hypertension, diabetes, and higher body mass index) in the districts of Fangshan (in southwestern periurban areas) and Pinggu (in northeastern periurban areas) compared with the average prevalence among residents of Beijing as a whole.^27^, ^28^, ^29^, ^30^, ^31^, ^32^ Geographic variations in AMI mortality might also be explained by the variations in health care across Beijing. The accessibility to health care is low in the southwestern and northeastern parts of Beijing, where AMI mortality is high.

After adjusting for changes in the prevalence of cardiovascular risk factors, we found that a relative increase in health care accessibility was associated with a relative decrease in AMI mortality. In this study, the increase in health care accessibility may be owing to the shortening of access time or an increase in the ratio of supply (hospital bed or health personnel) to demand (population). First, a shorter access time to hospitals among patients with AMI is related to decreased total ischemic time, which is critical to lowering the risk of death.^33^ In regard to the potential biological mechanism for this association, patients with AMI who have shorter ischemic times may have more myocardium that can be salvaged.^34^ Second, patients living in areas with a greater health care supply may be more likely to receive care in a hospital; prior studies have demonstrated that a greater health care resource supply is associated with lower mortality.^35^, ^36^

### Implications

The findings of our study have several implications. Previous research has reported that a lack of data on health inequalities and a subsequent lack of awareness of their existence are barriers to the design and implementation of policies to reduce inequalities.^37^ Our findings showed substantial geographic disparities in AMI mortality within Beijing, which can serve as a benchmark to examine these gaps in other rapidly developing megacities. Such information can be valuable for governments and health authorities to achieve the Health for All goal of the World Health Organization.^3^

Importantly, our findings showed that a relative increase in health care accessibility was associated with a decrease in AMI mortality. Understanding these associations is critical for identifying strategies to reduce the AMI mortality burden. This helps to support priority setting and to guide policymakers in allocating health care resources. Additionally, half of all AMI deaths occur out of the hospital,^2^ and thus, areas with high mortality should also focus on efforts such as prehospital education, including early recognition of AMI symptoms. Finally, a prior study observed similarities between the geographic patterns in AMI, stroke, and atrial fibrillation.^38^ Targeted allocation of health resources in areas identified as hotspots, which are initiated by decision makers, would be beneficial for controlling the total cardiovascular burden in megacities.

### Strengths and Limitations

To our knowledge, this was the first study to explore geographic variations in AMI mortality within rapidly developing megacities. Our study included citywide data on all AMI deaths in Beijing, which minimized the risk of selection bias. Furthermore, health care accessibility was measured using the enhanced 2‐step floating catchment area method, which incorporates the interaction between health care demand and supply and access time to provide a more comprehensive measure of health care accessibility. Finally, this study provides the first evidence of an association between relative increases in health care accessibility and decreases in AMI mortality at the township level.

This study had several limitations. First, our study included only administrative data, and it lacks comprehensive clinical data on AMI and treatment information. However, adjusting for cardiovascular risk factors and socioeconomic factors may have accounted for some of the variation in treatment among different areas. Second, there is the potential for misclassification caused by coding errors on causes of death. However, the diagnostic information in the BCDSS has been previously validated using the World Health Organization's Monitoring Trends and Determinants in Cardiovascular Disease criteria based on chart reviews, showing that the data quality is reasonably good.^39^ Third, this study, as with all observational evaluations, may be affected by unmeasured confounders or incomplete statistical adjustment for measured confounders that may have impacted the results and that future investigations should consider. Finally, township as the sampling unit means that the study design is prone to ecological fallacy. Thus, the study findings need to be interpreted with caution; we cannot determine causality owing to the ecological nature of the study.

## CONCLUSIONS

Although AMI mortality has decreased substantially during the past decade in Beijing, geographic inequalities in AMI mortality among townships are large and increasing. A relative increase in township‐level health care accessibility is associated with a relative decrease in AMI mortality. Policy efforts aimed at improving health care accessibility in areas with high mortality may help reduce AMI mortality and improve its geographic inequality in megacities. Further studies in other developing countries or regions are needed to validate our findings.

## Sources of Funding

This work is supported by grants from the National Natural Science Foundation of China (grant numbers 82073635 and 82103962), the Beijing Nova Programme Interdisciplinary Cooperation Project (grant number Z191100001119017), the Capital's Funds for Health Improvement and Research (grant number 2020‐1‐1051), and the Beijing Municipal Commission of Health (grant number 2021‐7). This work is supported by the Pathways to Equitable Healthy Cities grant from the Wellcome Trust (209376/Z/17/Z). For the purpose of Open Access, the author has applied a Creative Commons Attribution (CC BY) public copyright license to any author accepted article version arising from this submission.

## Disclosures

None.

## Supporting information

Data S1–S3
Tables S1–S5
Figures S1–S10
References [40, 41]
