# Supplementary material for: Geographic Variation in Mortality of Acute Myocardial Infarction and Association With Health Care Accessibility in Beijing, 2007 to 2018
Source: J Am Heart Assoc. 2023 Jun 10;12(12):e029769. doi: 10.1161/JAHA.123.029769 (PMC10356049; doi:10.1161/JAHA.123.029769)
Supplement: Supplementary file 1 — Data S1–S3 Tables S1–S5 Figures S1–S10 References [40, 41] [file JAH3-12-e029769-s001.pdf]

SUPPLEMENTAL MATERIAL

Supplemental Methods

Data S1. Validation of the Diagnosis of Acute Myocardial Infarction in the Beijing Cardiovascular Disease Surveillance System

The diagnoses in the Beijing Cardiovascular Disease Surveillance System have been previously validated, with "almost perfect" or "substantial" agreement (Kappa statistic range: 0.725 to 0.880) between the International Classification of Diseases code-based diagnoses in the system and clinical diagnoses in the original hospital charts. The positive predictive values of acute myocardial infarction diagnosis in the system were 94.4% and 87.9% in comparison with the criteria of the World Health Organization's Monitoring Trends and Determinants in Cardiovascular Disease (WHO-MONICA) project and the Third Universal Definition of acute myocardial infarction, respectively. The corresponding negative predictive values were 96.1% and 97.0%, respectively.[39]

Data S2. Enhanced Two-Step Floating Catchment Area Method

The enhanced two-step floating catchment area (E2SFCA) method is formulated as follows:[17]

Step 1: for each hospital (j), $$R_j = \frac{S_j}{\sum_{k\in[t_{k,j}\leq t_0]} D_k W(t_{k,j}, t_0)}$$

Step 2: for each township (i), $$A_i = \sum_{j\in[t_{i,j}\leq t_0]} R_j W(t_{i,j}, t_0)$$

Gaussian function, $$W(t_{i,j}, t_0) = \begin{cases} \frac{e^{-1/2\times(t_{i,j}/t_0)^2} - e^{-1/2}}{1 - e^{-1/2}}, & t_{i,j} \leq t_0 \\ 0, & t_{i,j} > t_0 \end{cases}$$

In the first step, for each hospital location (supply point) j, all township locations (demand point) k that are within the catchment area (a threshold of driving time (t_0)) of hospital j are searched and the supply-to-demand ratio R_j is calculated, where $t_{k,j}$ is the driving time between township centroid k and hospital j, and t_0 is the size of the catchment area of hospitals, i.e., the threshold of driving time for the range of hospital services. D_k is the demand size (population size) in township k whose centroid falls within the catchment area ($t_{k,j} \leq t_0$), and S_j is the capacity of supply at hospital j. $W(t_{k,j}, t_0)$ is the distance-decay function, and the Gaussian function is adopted to model the distance-decay effects, as suggested in existing studies,[17] which assumes that even within the same catchment area, people would give preference to closer hospitals than further ones, and the longer the driving time, the less likely the hospital is selected.

In the second step, for each township's location i, all hospital locations j that are within the catchment area ($t_{i,j} \leq t_0$) from location i are searched, and the supply-to-demand ratios of all hospitals located in the catchment area of township i are summed to obtain the accessibility A_i at township location i. $t_{i,j}$ is the driving time between township centroid i and hospital j. In this step, attenuation of the supply is also considered; therefore, the distance-decay function $W(t_{i,j}, t_0)$ is applied. A larger value of A_i indicates better health care accessibility at a location.

Chinese hospitals are classified as belonging to one of three levels: tertiary, secondary, or primary. Primary hospitals provide basic medical, prevention, rehabilitation, and health care services

to communities.[40] Secondary and tertiary hospitals provide medical treatments for patients with acute myocardial infarction (AMI). We further excluded specialized hospitals that do not have the ability to treat cardiovascular diseases, such as stomatology hospitals, cancer hospitals, psychiatric hospitals, and similar hospitals. Therefore, the E2SFCA model was used to measure the health accessibility of secondary and tertiary hospitals with the ability to treat cardiovascular diseases. Consistent with a previous related study on hospital accessibility in Beijing, the threshold time to measure accessibility was set to 60 minutes for secondary hospitals and 90 minutes for tertiary hospitals.[13] For the driving time between the hospital and township centroid, we computed the driving time in a motor vehicle along the road network between each township–hospital pair using a web mapping application program interface.[41] The number of hospital beds as the supply capacity for hospitals in the model, referring to previous studies,[17] was sourced from the Beijing Municipal Health Big Data and Policy Research Center of each hospital. The formula for the health care accessibility value is equal to the number of hospital beds per 1000 population.

Code executed by R

```
# Enhanced Two-Step Floating Catchment Area Method
# Step 1: calculate the supply-to-demand ratio R
library(tidyverse)
library(readxl)
data <-read_excel("F:/time_data.xlsx", sheet = "Sheet1")
data$time_cj <- data$time / 60
pop_data <- read_excel("F:/pop_data.xlsx", sheet = "Sheet1")
data <- merge(data, pop_data, by = c("ID", "Year"))
data$threshold <- ifelse(data$Hospital_level == "secondary", 60, 90)
data <- data %>% filter(time_cj <= threshold)
data$decay_1 <- exp(-0.5 * data$time_cj / data$threshold * data$time_cj / data$threshold) - exp(-0.5)
data$decay <- data$decay_1 / (1 - exp(-0.5))
data$total_pop_decay <- data$total_pop * data$decay
```

```
data_sum <- aggregate(data$total_pop_decay, by = list(data$weiyi_ID, data$Year), FUN = sum)
names(data_sum) <- c("weiyi_ID", "Year", "total_pop_decay")
bed_data <- read_excel("F:/bed_data.xlsx", sheet = "Sheet1")
data_sum <- merge(data_sum, bed_data, by = c("weiyi_ID", "Year"), all.x = TRUE)
data_sum$R_bed <- data_sum$Actual_beds / data_sum$total_pop_decay
data_sum <- data_sum[, !(names(data_sum) %in% c("total_pop_decay", "Actual_beds"))]
# Step 2: calculate the health care accessibility
data_2 <- read_excel("F:/time_data.xlsx", sheet = "Sheet1")
data_2$time_cj <- data_2$time / 60
data_2 <- merge(data_2, data_sum, by = c("weiyi_ID", "Year"), all.x = TRUE)
data_2$threshold <- ifelse(data_2$Hospital_level == "secondary", 60, 90)
data_2 <- data_2 %>% filter(time_cj <= threshold)
data_2$decay_1 <- exp(-0.5 * data_2$time_cj / data_2$threshold * data_2$time_cj / data_2$threshold)
- exp(-0.5)
data_2$decay <- data_2$decay_1 / (1 - exp(-0.5))
data_2$R_bed_decay <- data_2$R_bed * data_2$decay
data_2_sum <- aggregate(cbind(data_2$R_bed_decay), by = list(data_2$Year, data_2$ID), FUN = sum)
names(data_2_sum) <- c("Year", "ID", "R_bed_decay")
ID_data <- read_excel("F:/ID_data.xlsx", sheet = "Sheet1")
data_2_sum <- merge(data_2_sum, ID_data, by = c("ID", "Year"), all = TRUE)
for (i in 1:ncol(data_2_sum)){
   data_2_sum[,i][is.na(data_2_sum[,i])] <- 0
}
data_2_sum <- data_2_sum %>% rename(Access_bed = R_bed_decay)
data_2_sum$Access_bed_thou <- data_2_sum$Access_bed * 1000
data_2_sum <- data_2_sum[, !(names(data_2_sum) %in% c("Access_bed"))]
data_2_sum_wide <- reshape(data_2_sum, idvar = "ID", timevar = "Year", direction = "wide")
```

Data S3. Bayesian Spatial Model

The model is expressed as follows:

$$Deaths_d \sim Poisson(m_d . Population_d)$$

$$log(m_d) = \alpha_0 + b_d + h_d$$

where $Deaths_d$, $Population_d$, and m_d are the number of deaths, population size, and mortality rate, respectively, for township *d* for each period–sex–age group. α_0 is the common intercept. The terms b and h are components of the Besag–York–Mollié (BYM) model and are described in detail elsewhere.[20] Briefly, the random effects denoted by b were assumed to follow conditional autoregressive distributions that allow for smoothing rates of adjacent townships. The random effects denoted by h were assumed to follow independent mean zero normal distributions. In the BYM model, the estimated AMI mortality rate in each township is influenced by its own data and by the data of neighboring townships through spatially structured random effects with a conditional autoregressive prior distribution as well as globally through a spatially unstructured normal prior distribution.

Code executed by R

```
inits <- list (
   list(tau.b = 0.5, tau.h = 0.2, alpha =0),
   list(tau.b = 1.0, tau.h = 1.0, alpha =1.0))
parameters <- c("estimate")
library("R2OpenBUGS")
sim <- bugs(data, inits, parameters, model.file = "C:/PATH/ BUGS.txt",
n.chains = 2, n.iter = 15000, n.thin = 100, n.burnin=12000, codaPkg = FALSE, debug=
TRUE,DIC=TRUE, bugs.seed=8)
```

Code executed by BUGS

```
model {
        for (i in 1 : N) {

    # Likelihood
            Events [i] ~ dpois(estimate [i])
              estimate [i]<- rate [i]*population [i]
              log(rate [i]) <- alpha + b [i] + h [i]

    # prior on unstructured random effects
                h [i] ~ dnorm(0, tau.h)

        }

    # prior distribution for spatial random effects:

              b [1 : N] ~ car.normal(adj[], weights[], num[], tau.b)

    # Other priors:
        alpha    ~ dflat()
        tau.b    ~ dgamma(0.5, 0.0005)
        sigma.b <- sqrt(1 / tau.b)
        tau.h    ~ dgamma(0.5, 0.0005)
        sigma.h <- sqrt(1 / tau.h)
    }
```

Table S1. Townships Combined to Ensure Historically Stable Units of Analysis.

Townships	Name of Combined townships
Qingyuan township, Gaomidian township	Qingyuan/Gaomidian township
Xingjiekou township, Shichahai township	Xingjiekou/Shichahai township
Lugouqiao township, Lugouqiaodiqu township	Lugouqiao/Lugouqiaodiqu township
Huaxiang township, Xincun township	Huaxiang/Xincun township
Nanyuan township, Nanyuandiqu township,	Nanyuan/Nanyuandiqu township
Changxindian township, Changxindianzhen township	Changxindian/Changxindianzhen township
Huairou township, Quanhe township, Longshan township	Huairou/Quanhe/Longshan township
Houshayu township, Tianzhu township, Konggang township	Houshayu/Tianzhu/Konggang township
YiZhuang township, Yinghai township, Boxing township, Ronghua township	YiZhuang/Yinghai/Boxing/Ronghua township
Xiangyang township, Dongfeng township, Yingfeng township, Xingcheng township	Xiangyang/Dongfeng/Yingfeng/Xingcheng township
Dongxiaokou township, Tiantongyuannan township, Tiantongyuanbei township, Huoying township	Dongxiaokou/Tiantongyuannan/Tiantongyuanbei/Huoying township
Huilongguan township, Longzeyuan township, Shigezhuang township, Shahe township	Huilongguan/Longzeyuan/Shigezhuang/Shahe township

Lucheng township, Luyuan township, Zhongcang township, Yongshun township, Tongyun township	Lucheng/Luyuan/Zhongcang/Yongshun/Tongyun township

Table S2. Covariates and Definitions.

Covariates	Definitions
**Socioeconomic status**	
Proportion of the married population	The proportion of married population in the total population at the district level in 2010
Proportion of the population with a high school education or above	The proportion of population with a high school education or above in the total population at the township level in 2010
Per capita disposable income	Per capita disposable income at the district level from 2015 to 2018
Proportion of the unemployed	The proportion of unemployed population in the total population at the district level in 2010
**Cardiovascular risk factors**	
Prevalence of hypertension	In the survey of Beijing Chronic Disease and Risk Factors Surveillance, the proportion of population with hypertension in the surveyed population at the district level in 2008, 2011, 2014 and 2017
Prevalence of diabetes	In the survey of Beijing Chronic Disease and Risk Factors Surveillance, the proportion of population with diabetes in the surveyed population at the district level in 2008, 2011, 2014 and 2017

Prevalence of hypercholesterolemia	In the survey of Beijing Chronic Disease and Risk Factors Surveillance, the proportion of population with hypercholesterolemia in the surveyed population at the district level in 2008, 2011, 2014 and 2017
Prevalence of smoking	In the survey of Beijing Chronic Disease and Risk Factors Surveillance, the proportion of population with smoking in the surveyed population at the district level in 2008, 2011, 2014 and 2017
Percentage change in prevalence of hypertension	In the survey of Beijing Chronic Disease and Risk Factors Surveillance, the percentage change in prevalence of hypertension at the district level from 2008 to 2017
Percentage change in prevalence of diabetes	In the survey of Beijing Chronic Disease and Risk Factors Surveillance, the percentage change in prevalence of diabetes at the district level from 2008 to 2017
Percentage change in prevalence of hypercholesterolemia	In the survey of Beijing Chronic Disease and Risk Factors Surveillance, the percentage change in prevalence of hypercholesterolemia at the district level from 2008 to 2017
Percentage change in prevalence of smoking	In the survey of Beijing Chronic Disease and Risk Factors Surveillance, the percentage change in prevalence of smoking at the district level from 2008 to 2017

Table S3. Coefficient of Variation in the Mortality of Acute Myocardial Infarction by Township in Beijing, 2007–2018.

	2007–2009	2010–2012	2013–2015	2016–2018
Total	0.58	0.65	0.61	0.66
Men	0.55	0.61	0.58	0.62
Women	0.65	0.74	0.68	0.75

Table S4. Association Between Health Care Accessibility and Mortality of Acute Myocardial Infarction Among Beijing Townships*

	Model 1		Model 2		Model 3	
	β (95% CI)	*P* value	β (95% CI)	*P* value	β (95% CI)	*P* value
Total accessibility	−7.13 (−7.98 to −6.29)	< 0.001	−6.56 (−7.53 to −5.58)	< 0.001	−4.49 (−5.97 to −3.00)	< 0.001
PCI hospital accessibility	−10.34 (−11.59 to −9.09)	< 0.001	−9.16 (−10.54 to −7.78)	< 0.001	−6.43 (−8.73 to −4.13)	< 0.001
Non-PCI hospital accessibility	−17.11 (−19.43 to −14.79)	< 0.001	−15.78 (−18.69 to −12.88)	< 0.001	−9.10 (−12.45 to −5.75)	< 0.001

*Number of health care personnel was used as the health care supply capacity to calculate health care accessibility.

Model 1: not adjusted.

Model 2: adjusted for prevalence of hypertension + prevalence of diabetes + prevalence of hypercholesterolemia + prevalence of smoking.

Model 3: model 2 + proportion of married + proportion of population with high school education or above + per capita disposable income + proportion of unemployed.

Abbreviations: β, regression coefficient; CI, confidence interval; PCI, percutaneous coronary intervention.

Table S5. Association Between Percentage Changes in Health Care Accessibility and Percentage Change in Mortality of Acute Myocardial Infarction Among Beijing Townships*

	Model 1		Model 2		Model 3	
	β (95% CI)	*P* value	β (95% CI)	*P* value	β (95% CI)	*P* value
Per 10% increase						
Total accessibility	0.01% (−0.17% to 0.20%)	0.889	−0.68% (−1.02% to −0.35%)	< 0.001	−0.55% (−0.88% to −0.23%)	0.001
PCI hospital accessibility	–0.32% (−0.67% to 0.03%)	0.077	−0.24% (−0.59% to 0.12%)	0.191	−0.21% (−0.56% to 0.15%)	0.252
Non-PCI hospital accessibility	−0.21% (−0.33% to −0.10%)	< 0.001	−0.32% (−0.43% to −0.21%)	< 0.001	−0.25% (−0.35% to −0.14%)	< 0.001

*Number of health care personnel was used as the health care supply capacity to calculate health care accessibility.

Model 1: not adjusted.

Model 2: adjusted for percentage change in prevalence of hypertension + percentage change in prevalence of diabetes + percentage change in prevalence of hypercholesterolemia + percentage change in prevalence of smoking.

Model 3: model 2 + proportion of married + proportion of population with high school education or above + per capita disposable income + proportion of unemployed.

Abbreviations: β, regression coefficient; CI, confidence interval; PCI, percutaneous coronary intervention.

Figure S1. Townships in Urban Cores and Peri-Urban Areas of Beijing.

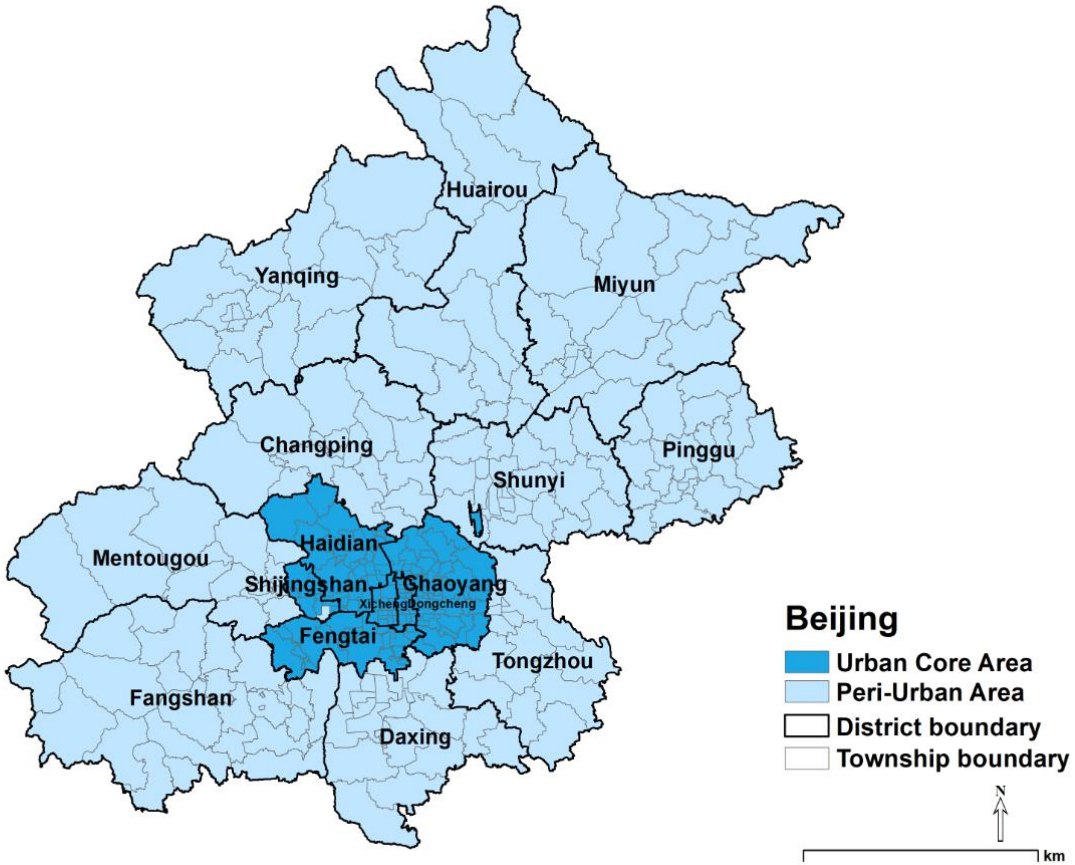

Figure S2. Deciles of Age-Standardized Mortality of Acute Myocardial Infarction in Beijing Residents Aged ≥ 35 Years at Township Level, 2010–2015.

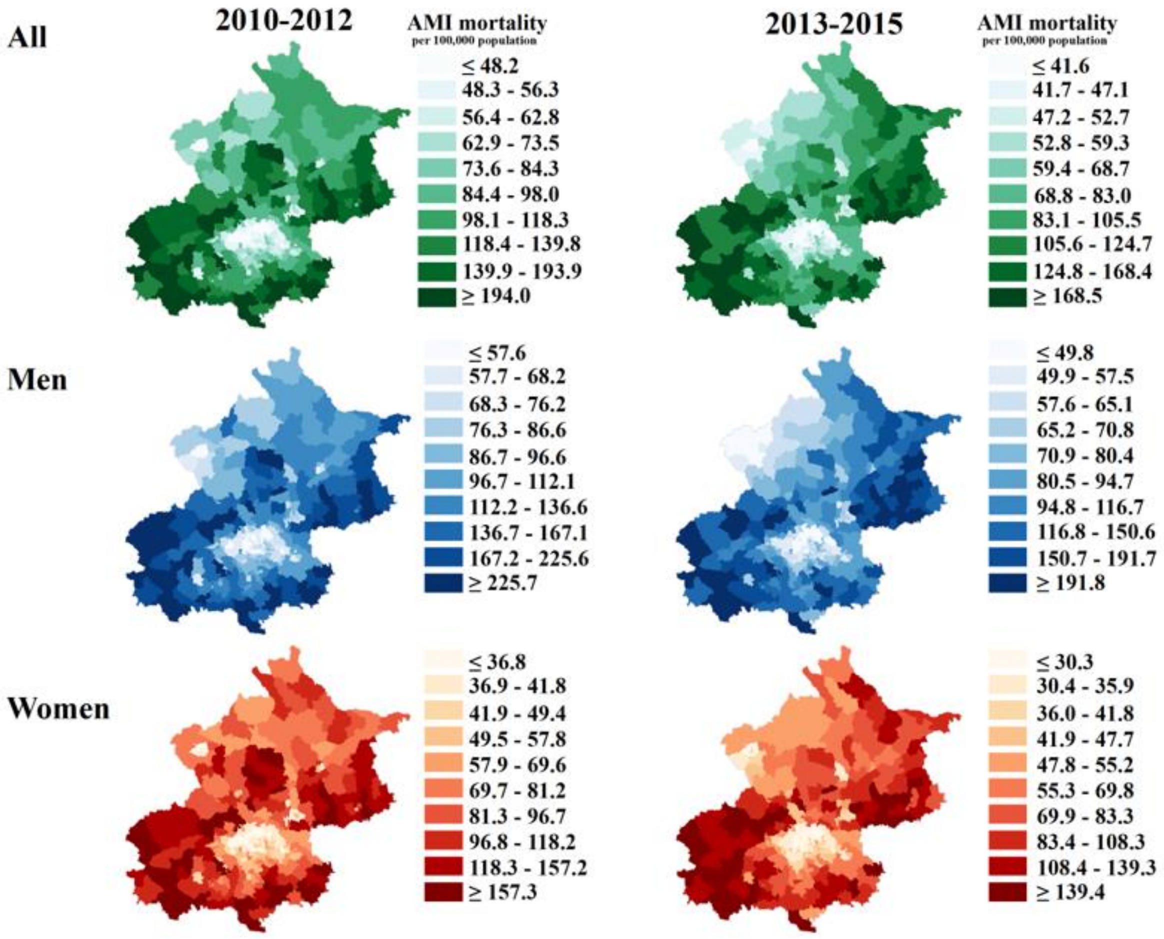

The green, blue, and red maps indicate the mortality in the total population, men, and women, respectively. A darker color indicates a higher mortality.

Figure S3. Proportion of the Number of Deaths by Age Groups at Township Level in Beijing, 2007–2018.

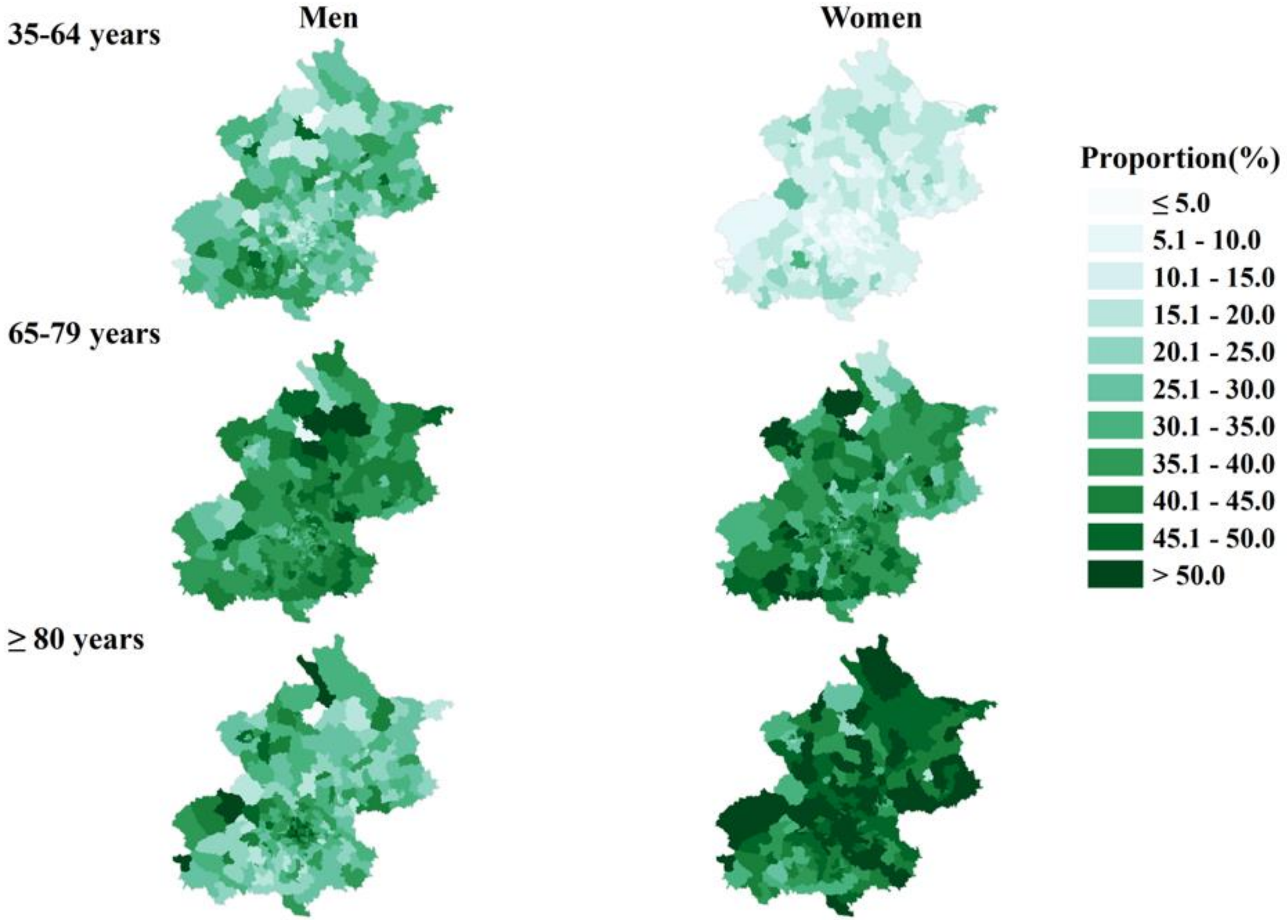

A darker color indicates a higher mortality.

Figure S4. Deciles of the Mortality of Acute Myocardial Infarction by Age–Sex Groups at Township Level in Beijing, 2007–2009.

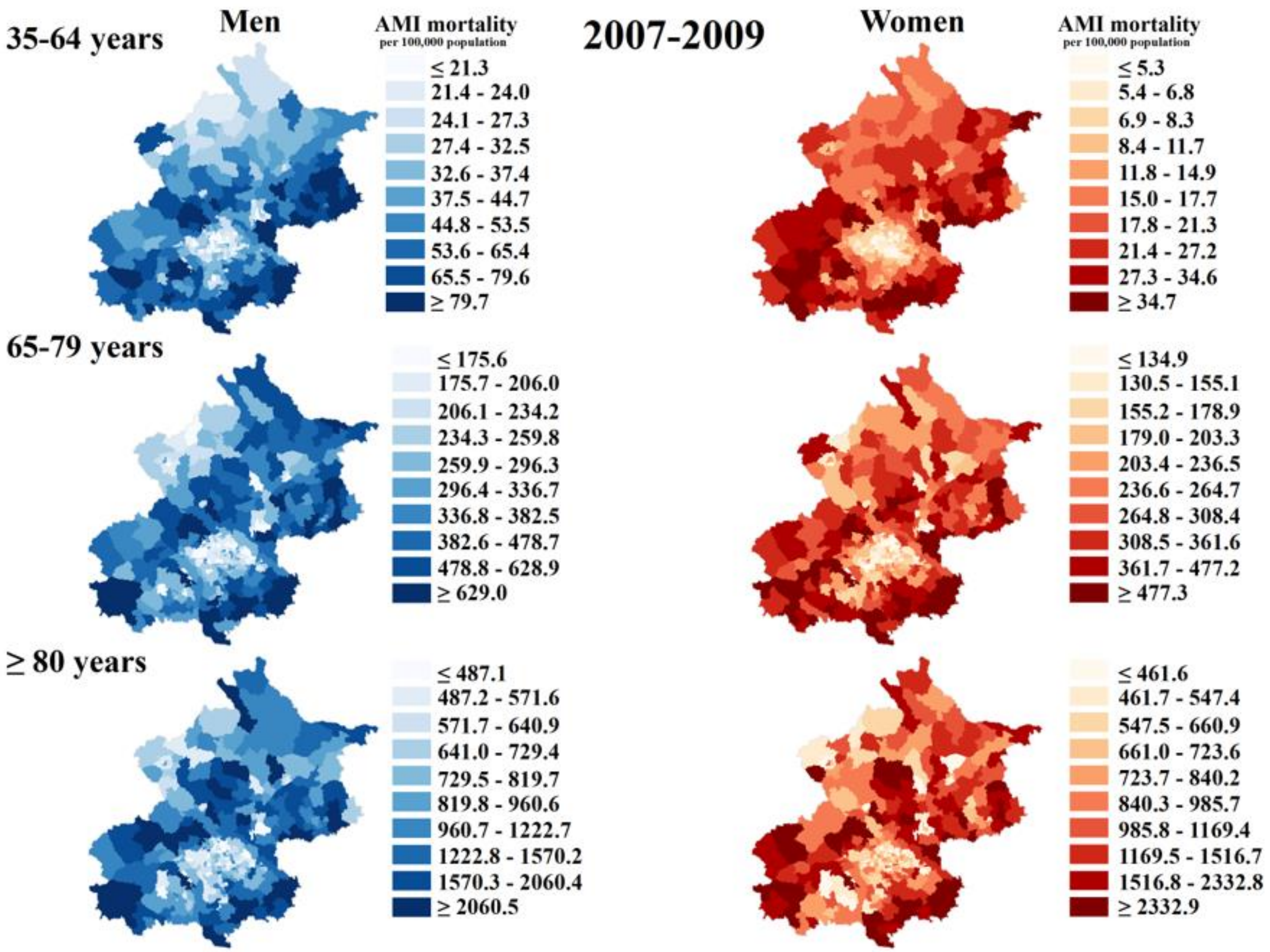

The blue and red maps show the acute myocardial infarction mortality in men and women, respectively. A darker color indicates a higher mortality.

Figure S5. Deciles of the Mortality of Acute Myocardial Infarction by Age–Sex Groups at Township Level in Beijing, 2010–2012.

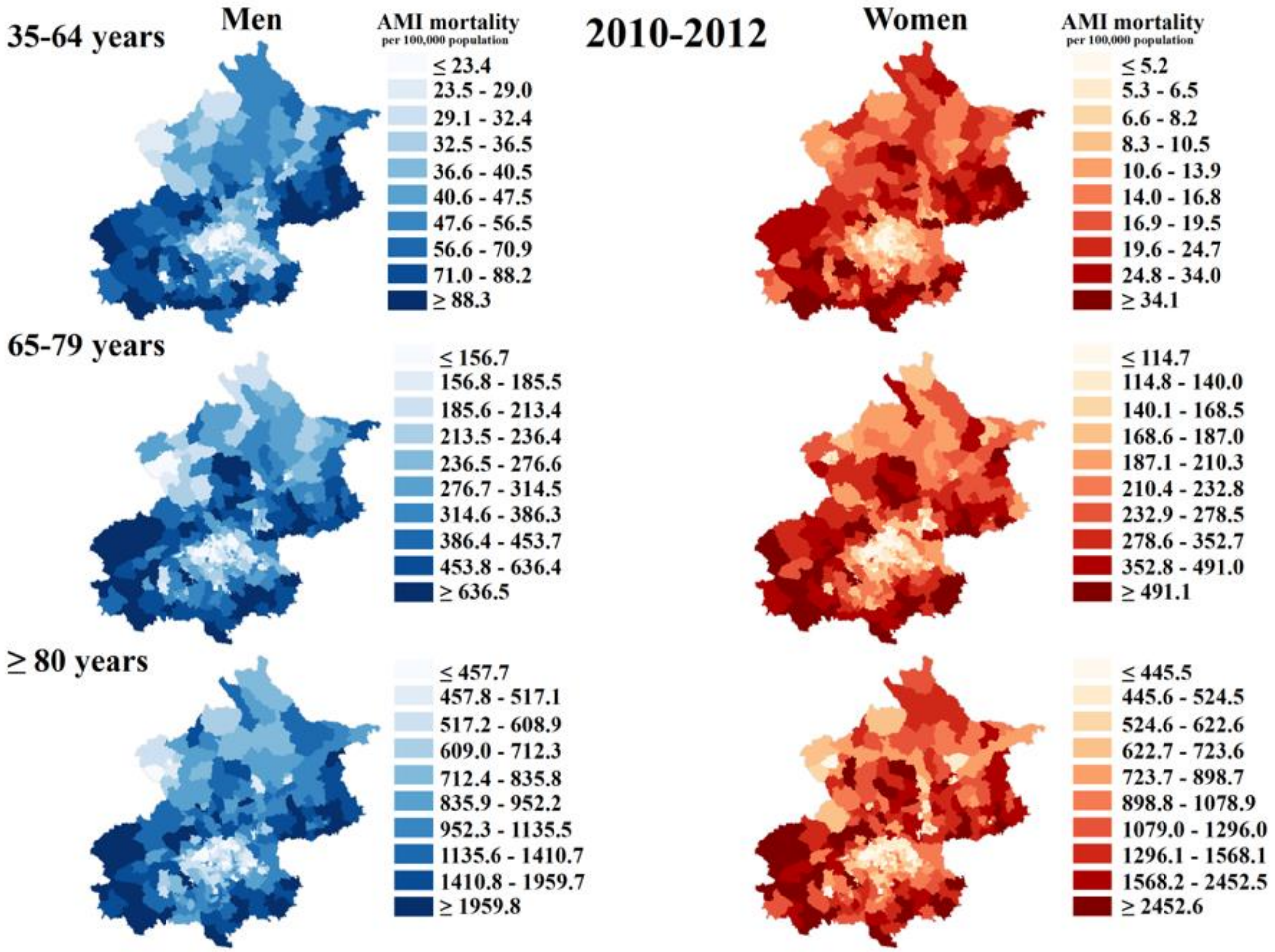

The blue and red maps show the acute myocardial infarction mortality in men and women, respectively. A darker color indicates a higher mortality.

Figure S6. Deciles of the Mortality of Acute Myocardial Infarction by Age–Sex Groups at Township Level in Beijing, 2013–2015.

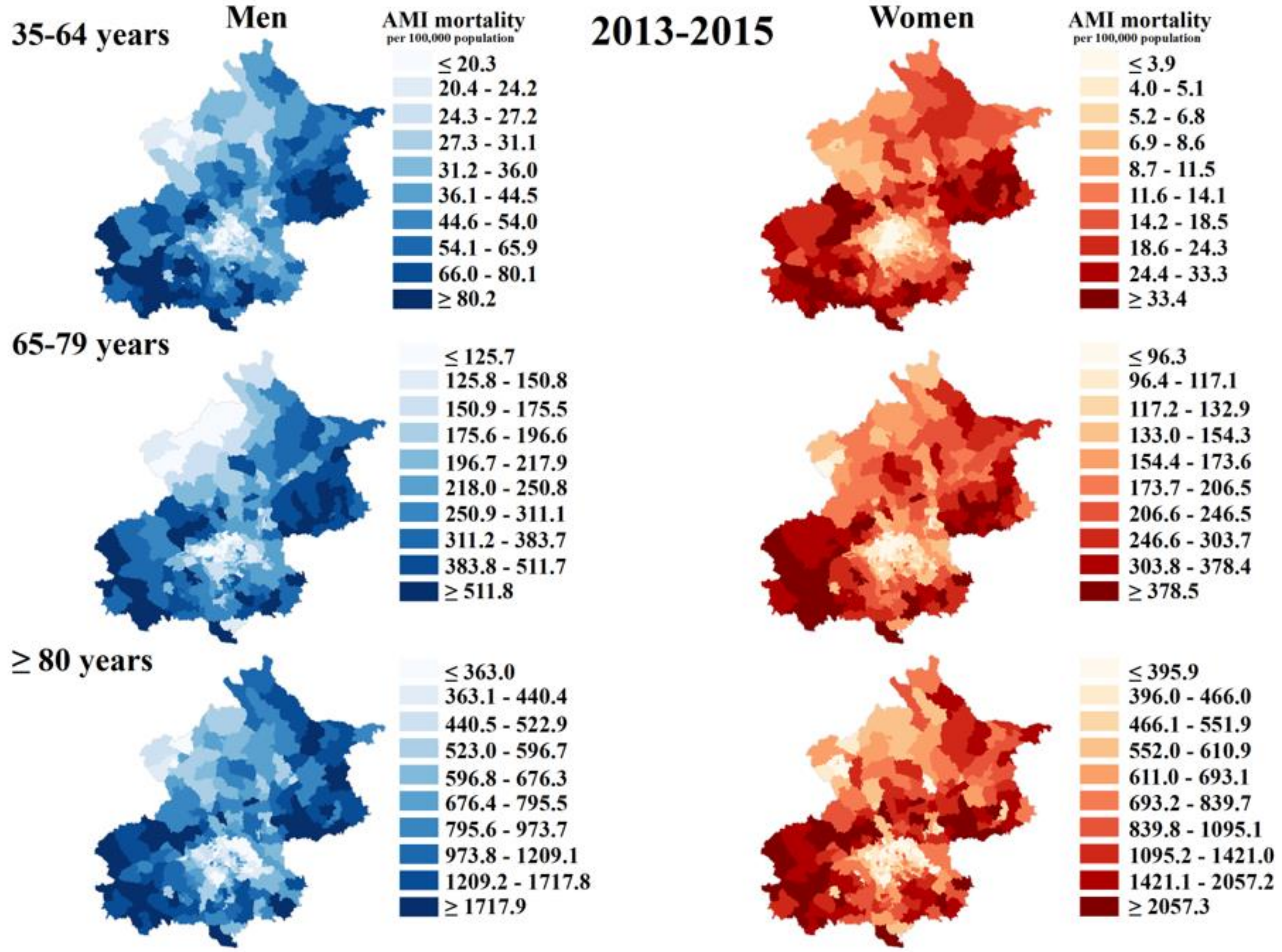

The blue and red maps show the acute myocardial infarction mortality in men and women, respectively. A darker color indicates a higher mortality.

Figure S7. Deciles of the Mortality of Acute Myocardial Infarction by Age–Sex Groups at Township Level in Beijing, 2016–2018.

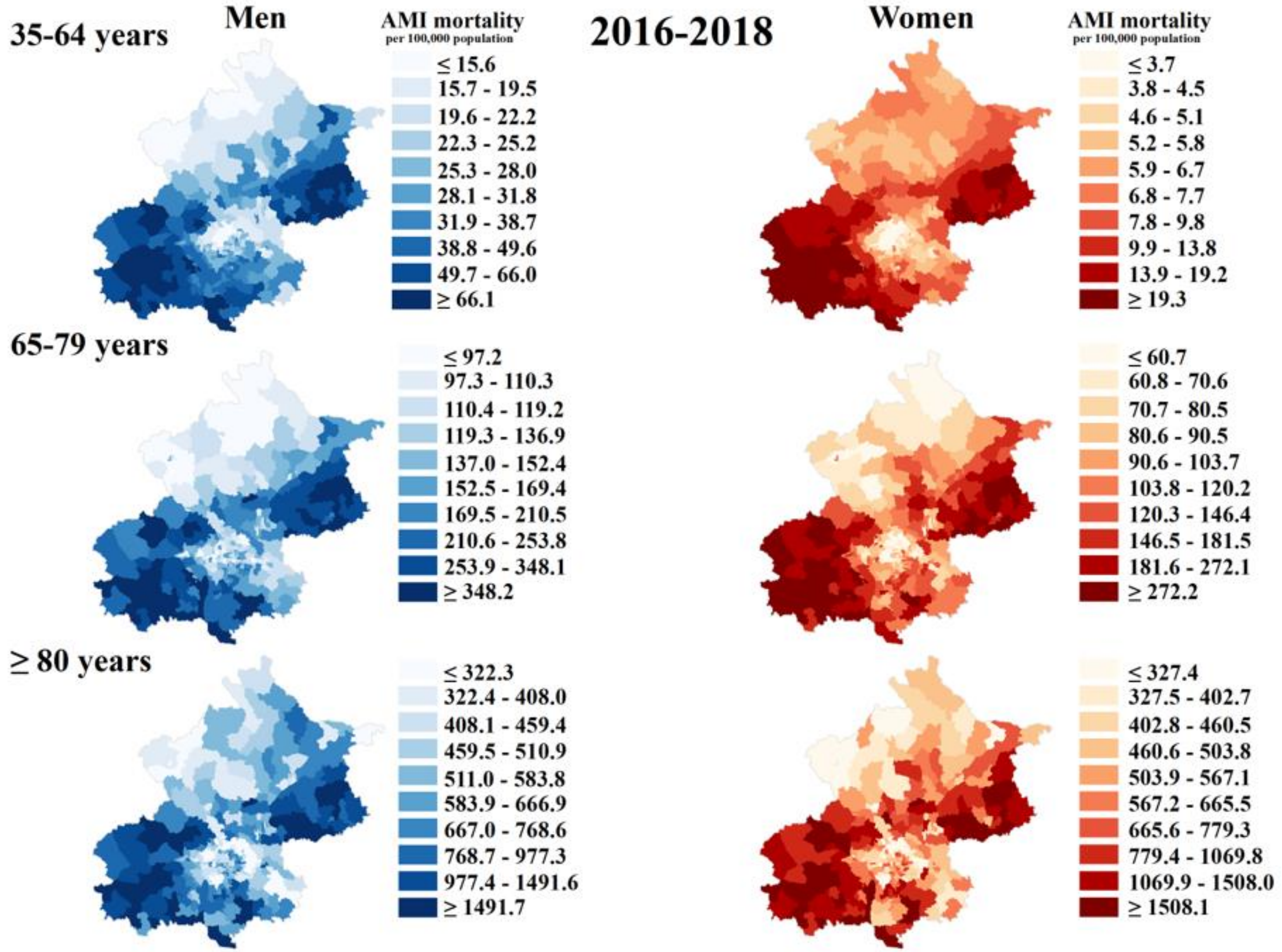

The blue and red maps show the acute myocardial infarction mortality in men and women, respectively. A darker color indicates a higher mortality.

Figure S8. Deciles of Health Care Accessibility in Beijing at Township Level, 2010–2015.

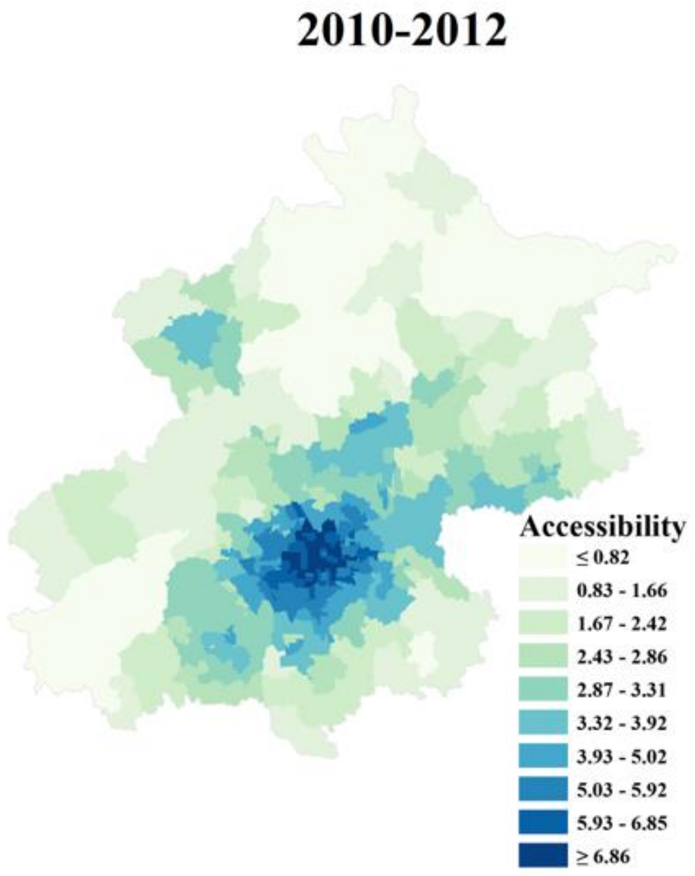

A darker color indicates a higher health care accessibility.

Figure S9. Deciles of Health Care Accessibility in Beijing by Percutaneous Coronary Intervention Hospital and Non-Percutaneous Coronary Intervention Hospital at Township Level, 2007–2018.

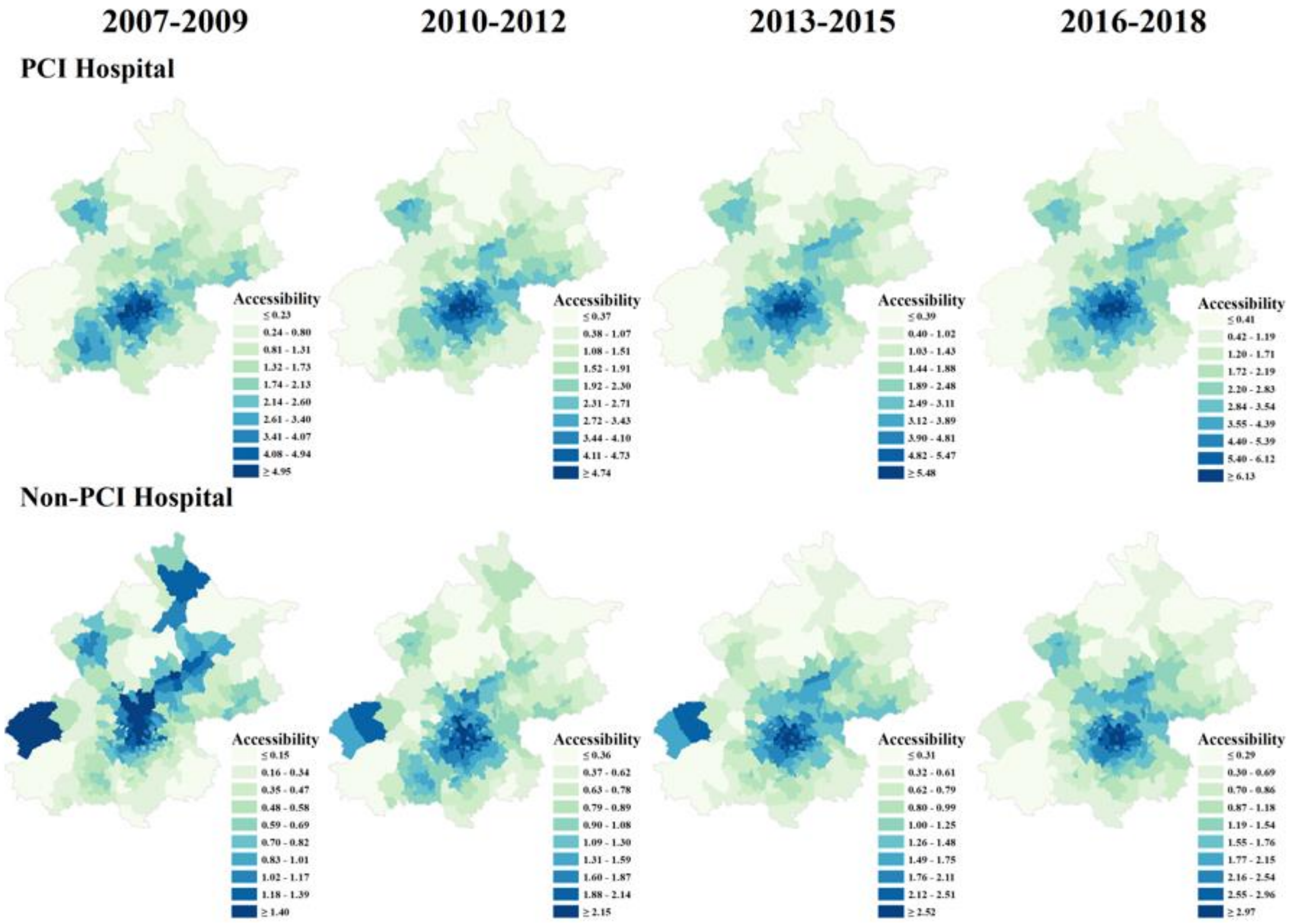

A darker color indicates a higher health care accessibility. Abbreviations: PCI, percutaneous coronary intervention.

Figure S10. Percentage Changes in Health Care Accessibility in Beijing by Percutaneous Coronary Intervention Hospital and Non-Percutaneous Coronary Intervention Hospital at Township Level, 2007–2018.

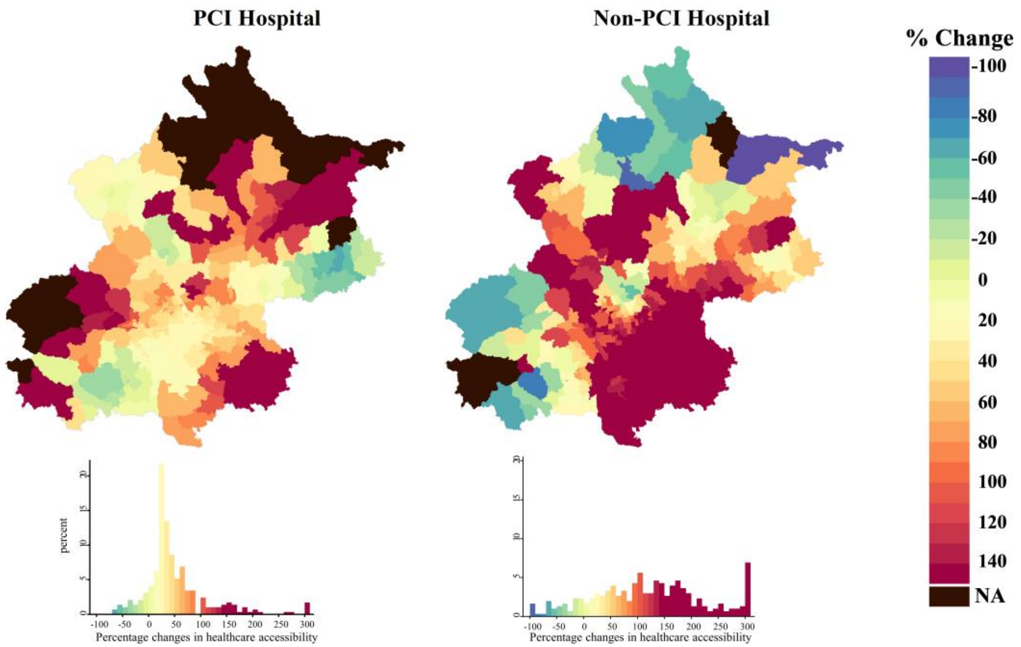

The percentage change shows the relative change in health care accessibility from the beginning of the study period (2007–2009) to the end of the study period (2016–2018). Blue indicates a decrease in health care accessibility, and red indicates an increase in health care accessibility. The histogram shows the distribution of percentage changes in health care accessibility for all townships.

NA indicates that the percentage changes in health care accessibility cannot be estimated, as the health care accessibility value of these townships is zero at the beginning of the study period (2007–2009). Abbreviations: PCI, percutaneous coronary intervention.
